# Advances in Fetal Surgery: A Narrative Review of Therapeutic Interventions and Future Directions

**DOI:** 10.3390/medicina61071136

**Published:** 2025-06-24

**Authors:** Antonia Varthaliti, Vasilios Pergialiotis, Marianna Theodora, Vasilios Lygizos, Maria Anastasia Daskalaki, Panos Antsaklis, George Daskalakis

**Affiliations:** 1st Department of Obstetrics and Gynecology, “Alexandra” General Hospital, National and Kapodistrian University of Athens, 80 Vasilissis Sofias Avenue, 11528 Athens, Greece; martheodr@gmail.com (M.T.); vlygizos@gmail.com (V.L.); anastasia.daskalaki00@gmail.com (M.A.D.); panosant@gmail.com (P.A.); gdaskalakis@yahoo.com (G.D.)

**Keywords:** fetal, interventions, intrauterine, myelomeningocele, congenital diaphragmatic hernia, fetal anemia, lower urinary tract obstruction, twin-to-twin transfusion syndrome

## Abstract

Fetal surgery has emerged as a viable option for the management of selected congenital anomalies that result in severe or lethal outcomes if left untreated until birth. Conditions such as spina bifida, urinary tract obstruction, congenital cystic adenomatoid malformation, diaphragmatic hernia, sacrococcygeal teratoma, and twin–twin transfusion syndrome have shown improved prognosis after in utero intervention, open, or fetoscopically. Despite significant advances in surgical methods and anesthesia, preterm labor remains a primary concern. Stem cell transplantation and in utero gene therapy are developing, and they have the potential to expand the treatment window, as they minimize maternal complications. Hematopoietic stem cell transplantation, which is based on the immaturity of the fetal immune system, is a promising treatment for inherited disorders. Although many procedures of fetal interventions are now established, their safety and efficacy must be ensured and this requires optimal patient selection and choice of appropriate timing for intervention, adherence to ethical principles, and continuous research. Therefore, a multidisciplinary team, including specialists in maternal–fetal medicine, pediatric surgery, anesthesiology, neonatology, psychosocial support, and bioethics, is essential to guide comprehensive, patient-centered care. Fetal surgery is an evolving field that offers hope for conditions previously considered untreatable before birth.

## 1. Introduction

Fetal therapy is a natural extension of fetal diagnosis. The development of diagnostic methods (such as ultrasound and fetal MRI) has led to the recognition and early diagnosis of congenital anomalies during pregnancy and therefore creates the possibility of timely and targeted intervention. The need for the development of fetal therapy arose from the observation that some, otherwise healthy, newborns exhibit congenital anomalies that prove to be life-threatening. When these conditions are diagnosed postnatally, the delay in intervention and the establishment of irreversible damage make their treatment less effective or even impossible and may lead to neonatal mortality or severe disability. Fetal surgery involves in utero invasive treatment of congenital anomalies which, during pregnancy, may lead to a serious deterioration of the condition of the fetus [1].

Fetal surgery, in less than five decades, has evolved from a pioneering idea into an internationally recognized, innovative, and technologically advanced surgical field. The need for intrauterine intervention arises mainly when an anatomical or functional anomaly is likely to worsen during pregnancy or when prenatal intervention is expected to offer a better prognosis compared to postnatal treatment. Thus, fetal surgery has emerged as one of the most dynamically evolving areas of maternal–fetal medicine, allowing in utero treatment of diseases associated with high perinatal morbidity and mortality, as well as serious long-term complications [2].

In addition, fetal anesthesia and the development of innovative minimally invasive tools have made intrauterine therapeutic intervention feasible and, in some cases, incorporated it into established clinical practice [3]. These interventions range from minimally invasive techniques, such as embryoscopic laser photocoagulation of placental anastomoses for the treatment of twin-to-twin transfusion syndrome, to open intrauterine surgery for the repair of congenital anomalies such as myelomeningocele [2].

However, fetal interventions have significant risks for both the mother and the fetus. Particularly in open procedures, maternal complications include preterm labor, rupture of membranes, and placental abruption, while the bioethical dilemmas associated with the simultaneous management of two patients with different needs and priorities are not negligible. Preoperative management includes thorough counseling and a multidisciplinary approach, while the selection of appropriate cases is based on strict criteria. The safety of the mother and the fetus is a primary concern when deciding whether to surgically intervene, while improving the prognosis is the next goal [4].

Fetal surgery includes a range of in utero interventions that aim to treat congenital anomalies before birth. There is open fetal surgery, minimally invasive fetoscopic procedures, and percutaneous techniques. Open fetal surgery involves a maternal laparotomy and uterine hysterotomy with direct exposure of the fetus, such as the open repair of myelomeningocele [5,6]. However, open procedures have maternal risks, such as uterine rupture and preterm labor [6]. Minimally invasive fetoscopic surgery utilizes small trocars to introduce a fetoscope and surgical instruments into the amniotic cavity under ultrasound guidance. It has become the standard of care for twin-to-twin transfusion syndrome via selective laser photocoagulation of placental anastomoses [7], fetoscopic spina bifida repair [8], and fetoscopic tracheal occlusion in congenital diaphragmatic hernia [9]. These techniques reduce maternal morbidity compared to open surgery, but they, as well, present high rates of preterm premature rupture of membranes [10]. Percutaneous fetal procedures, such as vesicoamniotic or thoracoamniotic shunting and intrauterine transfusions, are performed under ultrasound guidance without surgical incisions [11,12]. Risks include shunt displacement, infection, and fetal loss, particularly earlier in pregnancy. Fetal diagnosis, gestational age, maternal risk profile, and institutional expertise lead to the selection of the most appropriate technique [13].

In recent years, fetoscopy has also been developed, which aims to treat congenital anomalies, limiting the complications associated with open surgery, and it allows minimally invasive intrauterine intervention through endoscopic instruments. In some cases, the placement and manipulation of the instruments, as well as the ideal position of the fetus, can be facilitated by laparotomy; however, the applied technique is adapted for each case. Despite the promising advantages in terms of reducing maternal and fetal morbidity, fetoscopic surgery is still in under research, requiring more clinical studies and more cases [14]. Furthermore, its widespread adoption remains limited due to the need for highly specialized and excessively trained multidisciplinary teams, as well as high resource demands of fetal therapy centers [15].

The aim of this manuscript is to present an overview, summarized in Table 1, of the theoretical basis, the indications, approach techniques, the clinical results, and ethical implications of the most common procedures in modern fetal surgery, highlighting its importance as an evolving field of perinatal medicine.

## 2. Historical Evolution of Fetal Surgery

The history of fetal surgery is essentially a history of experimentation and innovation. Although fetal surgery is one of the most successful areas of innovation in history, it has been subject to controversy and criticism. The application of new, innovative treatments to pregnant women has led to the need to define new guidelines for ethical innovation and the conduct of clinical trials in humans.

Fetal surgery emerged in 1961, when William Liley performed the first percutaneous intrauterine blood transfusion to a fetus by blind intra-abdominal blood infusion, without ultrasound guidance. In 1964, Asensio and Adamsons described open hysterotomy for direct access to the fetus, for the purpose of blood transfusion for the treatment of Rhesus disease [1]. At the same time, it was observed that open procedures caused high morbidity and mortality. Hysterectomy was abandoned, and it was more than a decade before the possibility of open fetal surgery was explored again [16].

The origins of modern fetal surgery can be traced to the University of California, San Francisco (UCSF), with Michael Harrison, who is considered the father of fetal surgery, as the central figure. In 1969, as a surgical resident at Massachusetts General Hospital, Harrison assisted Dr. Hardy Hendren in a repair operation for a congenital diaphragmatic hernia. Despite surgical success and postoperative care, the newborn died the next day from pulmonary hypoplasia. The disappointing outcome inspired his first “crazy idea”: to repair the defect before birth. This experience defined his career, led him to UCSF, and made CDH a focal point for the development of fetal surgery [17].

Subsequently, other congenital conditions were identified as candidates for intrauterine intervention, with the aim of either protecting the life of the fetus or preventing permanent damage. These interventions may aim either at anatomical correction of the malformation or at inhibiting the progression of the disease, so that final repair can occur postnatally. The criteria for the application of intrauterine therapeutic interventions were summarized in a consensus document drafted by the International Fetal Medicine and Surgery Society (IFMSS), under the guidance of Harrison in 1982 [18]. A similar dynamic was observed in Europe, with the important contribution of the group of Charles Rodeck at King’s College Hospital and, subsequently, of Kypros Nicolaides at the Harris Birthright Centre in London. When King’s College, as a major training center in fetal and maternal medicine, adopted embryoscopy in the 1990s, the technique spread rapidly throughout Western Europe [19]. The history of fetal surgery was established through experimental research in animal models (such as sheep and monkeys), with the aim of developing safe techniques for accessing the uterus and experimentally repairing congenital anomalies [20].

The following are the most common pathological conditions for which prenatal treatment through intrauterine intervention is indicated. These are congenital anomalies with high perinatal morbidity and/or mortality, for which intervention in the fetus can significantly improve the prognosis and future functionality of the newborn.

## 3. Fetal Anemia

Fetal anemia is a pathological decrease in fetal hemoglobin or hematocrit concentration compared to normal values for the corresponding gestational age. According to the literature, the diagnosis is made when hemoglobin is 2–7 g/dL below the mean normal value for gestational age (moderate anemia) or >7 g/dL below normal (severe anemia). In addition, a fetal hematocrit <30% is considered indicative of anemia in advanced pregnancy [21].

Early and accurate diagnosis is crucial in high-risk fetuses, as intrauterine transfusion can significantly improve prognosis and survival. Understanding the immunological and non-immunological mechanisms of fetal anemia, as well as advances in high-resolution ultrasound imaging, have led to significant developments in both the technique and protocols of intrauterine transfusion. Despite routine administration of prophylactic anti-D immunoglobulin during pregnancy, Rhesus alloimmunization and hemolytic disease of the fetus and newborn (HDFN) continue to occur, due to a multitude of pathophysiological and clinical factors.

### 3.1. Causes of Fetal Anemia

Fetal anemia can be a result of immunological and non-immunological causes. Immunological causes include maternal alloimmunization against fetal red cell antigens, most commonly anti-D, anti-Kell (K1), anti-c (RH4), anti-Duffy, and anti-Kidd, leading to hemolysis and hemolytic disease of the fetus and newborn. Non-immunological causes include infections, such as parvovirus B19 and cytomegalovirus infection, congenital syphilis, congenital hematological disorders (e.g., thalassemias, congenital aplastic anemias), severe feto-maternal hemorrhage, and complications of monochorionic twin pregnancies (e.g., TTTS, TAPS). Other rare genetic causes of fetal anemia are disorders of red blood cell production (e.g., Diamond-Blackfan anemia, Fanconi anemia), erythrocyte enzyme disorders (e.g., glucose-6-phosphate dehydrogenase deficiency, pyruvate kinase deficiency), structural abnormalities of red blood cells (e.g., hereditary spherocytosis and elliptocytosis), lysosomal storage disorders (e.g., Gaucher disease, Niemann-Pick disease, mucopolysaccharidosis type VII), and myeloproliferative syndromes such as leukemias. Early recognition and understanding of the cause is critical for timely therapeutic intervention and improved perinatal outcome.

### 3.2. Diagnosis of Fetal Anemia

Prenatally, the peak systolic velocity of the middle cerebral artery (MCA-PSV) is the most reliable non-invasive indicator. Measurement of MCA-PSV, using Doppler, is an established prenatal screening tool. The presence of moderate to severe fetal anemia is documented when the fetal MCA-PSV exceeds 1.5 MoM, between the 18th and 35th weeks of gestation. Measurement of MCA PSV shows a sensitivity of 86% and a specificity of 71% for the diagnosis of fetal anemia. The measurement is performed in a cross-sectional view of the brain, with visualization of the ventricles, the septum pellucidum, the greater part of the sphenoid bone, and the circle of Willis, and the measurement is obtained by selecting the MCA closest to the probe, as close as possible to its point of origin from the internal carotid artery, and maintaining a Doppler approach angle as close as possible to 0°. In some cases, fetal anemia may be accompanied by fetal hydrops, which is defined as the presence of two or more pathological fluid collections in the fetus. These collections include ascites, pleural and pericardial collections, as well as generalized skin edema, which is characterized by a skin thickness greater than 5 mm, and are found during ultrasound [22,23].

In cases of positive MCA-PSV or presence of fetal hydrops, a fetal blood sample is obtained by cordocentesis for measurement and determination of hematocrit and hemoglobin, direct Coombs test, blood group, reticulocyte, biochemistry (e.g., bilirubin), and karyotype, if required. Fetal blood sampling is performed from the 18th to the 35th week of gestation. Fetal anemia is classified based on hemoglobin deficiency into moderate (deficit 2–7 g/dL) and severe (>7 g/dL). In cases of hydrops, the hematocrit is usually ≤15% [24].

### 3.3. Fetal Red Blood Cell Transfusion

Intrauterine fetal blood transfusion is a therapeutic fetal intervention in which, specially processed and prepared donor red blood cells are administered intravenously into the umbilical cord, under ultrasound guidance. This technique, which was first applied in 1963, continues to be the treatment of choice for severe fetal anemia to this day. The aim of intrauterine fetal transfusions is to improve the oxygen-carrying capacity of the fetal blood, through the intravenous administration of high-concentration O Rh-negative red blood cells, which replace hemolyzed fetal cells and prevent the development of hydrops and the increased perinatal mortality. In addition, it aims to suppress excessive fetal erythropoiesis, to reverse already developed hydrops, as well as to prolong pregnancy until delivery of a healthy, non-edematous newborn. Finally, timely and effective intrauterine transfusion contributes to reducing the need for postnatal blood exchange and prolonged phototherapy [25].

Blood transfusion to the fetus is indicated when the MCA PSV exceeds 1.5 MoM or if hydrops is present and anemia is considered the likely cause. There must also be laboratory confirmation by obtaining fetal blood and finding a fetal hematocrit <30% or hemoglobin <10 g/dL, or a hemoglobin deficit >7 g/dL below the mean value for gestational age. Intrauterine transfusion is usually recommended from 18 to 35 weeks of gestation.

Transfusion is usually performed intravascularly, and the main anatomical access sites include the site of the cord insertion to the placenta, the intrahepatic vein, or a free cord loop. Early in the second trimester, if the umbilical vein is too thin to allow the needle to enter, fetal intraperitoneal transfusion is performed. In cases of hydrops, peritoneal absorption is reduced, and some prefer to transfuse into both the peritoneal cavity and the umbilical vein. The donor blood required for the intrauterine transfusion must be obtained from a specially prepared O Rh D negative blood transfusion unit. Such units are usually located in tertiary hospitals, research and educational centers, oncology institutions, or specialized blood banks. At the beginning of the procedure, a blood sample is drawn from the fetus by cordocentesis to measure the fetal hematocrit and the value is recorded for dose calculation. Before the transfusion, a muscle relaxant such as vecuronium can be administered intramuscularly to the fetus using a 22G needle to minimize fetal movement. Percutaneous access to the predetermined target is performed using a 20G needle, under ultrasound guidance. After a successful entry into the umbilical cord, a blood sample is taken for hematocrit measurement and a complete blood count is obtained to exclude other hematological pathologies [26].

To accurately determine the required volume of blood for the transfusion, the initial fetal hematocrit, the fetoplacental blood volume, the donor hematocrit, and the desired hematocrit (target: 45–50%) are taken into account. The formula commonly used to calculate the required volume is as follows:

Transfusion volume = Fetoplacental blood volume × (Desired Hct − Fetal Hct before transfusion)/(Donor Hct − Desired Hct) [19].

The fetoplacental volume is calculated based on the estimated fetal weight (EFW) according to the ultrasound calculation, with the following formula: Fetoplacental blood volume = EFW × 0.14.

This calculation is based on either fetal weight or gestational age, with the latter not being affected by the presence of fetal hydrops. For the severely anemic fetus between 18 and 24 weeks of gestation, a smaller volume of blood is initially transfused and a second transfusion is scheduled approximately two days later. After the volume of blood to be transfused has been accurately calculated, the calculated volume of blood is transfused under strict aseptic conditions. Throughout the transfusion, fetal movement is closely monitored and the heart rate is recorded, with attention to the appearance of fetal bradycardia. After the transfusion and administration of the total calculated volume, 2 mL of normal saline is injected through the same needle and a new fetal blood sample is taken to measure the hematocrit after transfusion. Post-transfusion monitoring is performed by assessing the hematocrit, which will determine whether further transfusions are needed. At the same time, fetal heart rate, MCA PSV, and myocardial function index are recorded [27].

Weekly monitoring of MCA-PSV is then recommended for early recognition of recurrent anemia. In non-hydropic fetuses, the target hematocrit is 40–50%. Subsequent transfusions are given every 2 to 4 weeks, depending on the hematocrit. After the first transfusion, the threshold for MCA-PSV indicating severe anemia increases to 1.70 MoM, as opposed to 1.50 MoM before transfusion. This is probably due to the lower mean red cell hemoglobin of adult donors. After transfusion, fetal hematocrit decreases by approximately 1% per day, more rapidly if hydrops is present.

To prepare the mother for fetal transfusion, corticosteroid coverage for lung maturation is recommended at gestational age greater than 26 weeks, and should be given 48 h before transfusion. There is no evidence for the routine use of prophylactic antibiotics.

A successful intrauterine transfusion protocol aims to deliver a neonate ≥37 weeks gestation without transfusion or phototherapy. In general, if the last transfusion was performed between 34 and 35 weeks, delivery can be scheduled 3 to 4 weeks later. Induction of labor may be recommended if there are no obstetric contraindications. Delivery should occur in a hospital with a neonatologist available.

### 3.4. Complications of Fetal Red Blood Cell Transfusion

Complications that may occur during or after intrauterine transfusions include fetal bradycardia in 5–10% of cases, while local events in the umbilical cord, such as rupture, spasm, hematoma occlusion, excessive bleeding or umbilical artery thrombosis, can lead to hemodynamic instability of the fetus. Other complications include fluid overload, chorioamnionitis, preterm premature rupture of membranes (<0.3%), iatrogenic preterm labor that may be accompanied by neonatal asphyxia or death, while intrauterine fetal loss is reported at rates ranging between 0.9 and 4.9%. If transfusion is required before 20 weeks, the probability of miscarriage reaches 15%. In fetal hydrops, survival is approximately 80%. When hydrops resolves after intrauterine transfusion, survival reaches 95%, compared to <40% when hydrops persists. Regarding long-term neurodevelopmental outcome, it has been found to be normal in 95% of children evaluated at a mean age of 8.2 years. Cerebral palsy, severe developmental delay, and bilateral deafness were diagnosed in 2%, 3%, and 1% of children, respectively [12]. The perinatal loss before 20 weeks of gestation has been reported at approximately 24%, compared with 8% in cases where the first transfusion was performed after 20 weeks [28], while procedure-related fetal demise (occurring within 48 h) was about 7%, with a similar 8% neonatal mortality at 18–32 weeks of gestation (median 25.2 weeks) [29].

## 4. Twin-to-Twin Transfusion Syndrome, Twin Anemia-Polycythemia Sequence, and Twin Reversed-Arterial Perfusion

**Figure 1:**
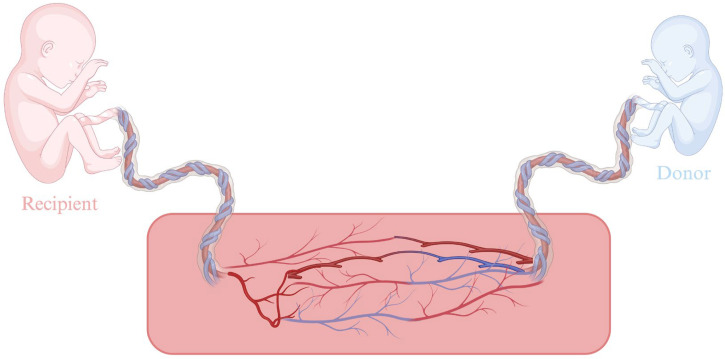
Placental anastomosis in TTTS. Created in BioRender. Varthaliti, A. (2025). https://BioRender.com/ym3pg5m.

Monochorionic twins are characterized by a shared placenta with vascular anastomoses in the chorionic petal, which allow bidirectional blood flow between them. This unique circulatory connection predisposes to serious complications, such as imbalance between the two fetuses, which may affect body size, amniotic fluid volume, placental perfusion, or even morphological abnormalities, as shown in Figure 1. Clinical manifestations include selective intrauterine growth restriction, intrauterine death, perinatal mortality, and hemodynamic syndromes such as twin-to-twin transfusion syndrome (TTTS), Twin Anemia-Polycythemia Sequence (TAPS), and twin reversed-arterial perfusion (TRAP). TTTS occurs in approximately 15% of monochorionic pregnancies. It is a hemodynamic asymmetry, secondary to unequal blood flow through the placental vascular anastomoses [30].

Today, the most frequently performed intrauterine procedure is undoubtedly embryoscopic laser photocoagulation [yttrium–aluminum–garnet (Nd:YAG; wavelength 1064 nm, 50–100 W or diode (940 nm, 20–60 W)] of placental anastomoses in twin-to-twin transfusion syndrome, a complication of monochorionic pregnancies characterized by unequal blood distribution between the fetuses through vascular anastomoses in the placenta. Severe cases, if left untreated, often lead to the loss of one or both fetuses. Laser photocoagulation is performed through a single-entry point and aims to destroy these vessels, thus resolving the underlying cause of the disorder. The procedure is performed between the 16th and 26th weeks of gestation in monochorionic-diamniotic pregnancies with TTTS stage II to IV. The technique involves inserting the embryoscope into the amniotic sac of the recipient fetus and positioning it over the vascular isomerism of the placenta, which is located between the entry sites of the umbilical vessels of the two fetuses. During the procedure, the transplacental arteriovenous anastomoses are visually identified on the surface of the placenta and subjected to selective laser photocoagulation, which aims to inactivate each vascular anastomosis. Due to its documented effectiveness and relatively low complication rate, it is now considered the treatment of choice. The procedure is usually performed under regional or light general anesthesia. At the end of the procedure, amniotic fluid is aspirated, with the aim of reducing its amount and lowering intrauterine pressure. In addition, prophylactic antibiotics are administered intraamniotically [31].

However, in approximately 15–30% of cases, after photocoagulation, vascular anastomoses that have not been identified may remain. The persistence of residual anastomoses post-laser is usually attributed to both technical challenges (incomplete placental mapping) and the complexity of placental vascular architecture, which results in difficulty inthe recognition of the anastomoses. This can lead to the recurrence of the fetal–fetal transfusion syndrome, at a rate of 14%, or to the development of a variant of it, known as TAPS, at a rate of 13%, which is characterized by a significant difference in hemoglobin between the two twins without obvious hypervascularization. The application of the Solomon technique, which involves cauterizing the chorionic surface of the placenta along the vascular anastomoses, from one end to the other, has been shown to reduce the frequency of recurrences and residual anastomoses. However, even after the application of the Solomon method, residual vascular anastomoses may lead to the recurrence of TTTS or TAPS at a rate of up to 3.9% [32,33,34,35].

Information to parents should reflect both the chances of success and the potential risks of the treatment. Without treatment, perinatal mortality in severe cases of TTTS ranges from 70 to 100%. After laser treatment, perinatal mortality is reduced to 30–50%. According to studies, the survival rate of both fetuses is almost 70%, while at least one fetus survives in more than 90%. Ischemic brain lesions (such as periventricular leukomalacia cysts or grade III or IV hemorrhage) have been recorded in approximately 2% of cases, and cerebral palsy has been reported in 5% of children surviving after laser treatment for TTTS [36].

Technical complications of the procedure include premature rupture of fetal membranes in up to 25% of cases, placental abruption in 8%, vascular injuries in 3%, as well as amniotic band syndrome resulting from laser-related membrane injury. The majority of pregnancies treated with laser for TTTS result in delivery before 34 weeks of gestation [37].

Other complications of monochorionic pregnancies, such as TRAP or selective intrauterine growth restriction, when severe, can lead to fetal death that cannot be prevented by embryoscopy. The death of one fetus in a monochorionic pregnancy directly endangers the surviving fetus due to vascular anastomoses and rapid hemodynamic changes. To avoid burdening the healthy twin, selective reduction with radiofrequency cord occlusion is applied. The procedure is performed under ultrasound guidance. A 17 or 19G RFA instrument is placed at the base of the twin’s umbilical cord where selective reduction will be performed, through the mother’s abdominal wall. The procedure is ideally performed after the 20th week of pregnancy. Complications include premature rupture of membranes and premature labor [38].

## 5. Myelomeningocele

Spina bifida is the most common congenital anomaly of the central nervous system compatible with life. The most common form is myelomeningocele, which despite the reinforcement of the diet with folic acid during pregnancy has an incidence in the United States of 3.4 per 10,000 births. It is caused by the failure of closure of the neural tube during the first four weeks after conception. It consists of a cyst containing the spinal cord, nerve elements, and cerebrospinal fluid, which remain exposed to the amniotic fluid. Open neural tube malformation leads to pathological development of the central nervous system (CNS), resulting in hydrocephalus, anterior projection of the cerebellum and damage to the nerve elements due to amniotic fluid toxicity, which is associated with significant morbidity and mortality long term. Although approximately 75% of patients survive to adulthood, the survival rate in the first year of life ranges between 88% and 96%. The majority of patients with myelomeningocele (≥80%) require placement of a ventriculoperitoneal drainage valve for hydrocephalus, mainly in high lesions, with a risk of complications such as infection and obstruction. More than 75% have Arnold–Chiari II malformation, with possible manifestations of apnea, dysphagia, or tetraparesis. The severity of neurological disability in the lower extremities is correlated with the level of the spinal cord lesion. Urinary and fecal incontinence are common, as well as urinary tract infections, reflux and upper urinary tract distension [39].

In neonates with prenatal diagnosis, the lesion is usually surgically repaired during the neonatal period, although the lesions are usually progressive during pregnancy, with progressive loss of lower limb mobility, worsening hindbrain prolapse, and increasing hydrocephalus, and are irreversible, even after early postnatal surgical repair. Thus, the need for prenatal intrauterine therapy emerged. The main concern of intrauterine repair is to maintain the integrity of the neural elements by protecting the exposed neural plate from further damage. The first intrauterine repair of myelomeningocele via hysterotomy in humans was performed in 1997, and by 2003, over 200 fetuses had been operated on. Initial data showed a dramatic improvement in brain prolapse compared with the control population, but also an increased maternal risk, with complications such as preterm delivery, uterine rupture, and increased perinatal morbidity and mortality. Intrauterine surgery is a highly invasive intervention and is currently only applicable in severe fetal cases, where intrauterine repair could favorably alter the natural course of the disease, which would otherwise lead to neonatal death or severe disability. The significant risks to the mother and fetus highlighted the need for careful evaluation of the appropriateness of these interventions [40].

### 5.1. Open Intrauterine Surgical Repair of Myelomeningocele

The open intrauterine repair of myelomeningocele is performed under combined general and epidural anesthesia, with laparotomy (low transverse or vertical) and hysterotomy in the uterine vault. Before the hysterotomy, an ultrasound is performed during which the position of the placenta and the fetus is determined. The fetus must be in a cephalic projection. When the fetus is in breech presentation, cephalic version is performed to convert the fetus into cephalic presentation. After determining the optimal hysterotomy point, two full-thickness stabilization sutures are placed approximately 1 cm apart in an area free of placenta, umbilical cord, or fetal limbs under ultrasound guidance. The hysterotomy is performed with diathermy. Atraumatic intestinal forceps are initially placed at the hysterotomy line and then a stapler is used. This creates a clean field of 6–8 cm in which the fetal myelomeningocele is immediately accessible. Warmed Ringer’s solution is injected through the incision to maintain warmth and buoyancy, while intraoperative echocardiographic monitoring is performed throughout to check fetal cardiac function and adjust the parameters of the procedure [41].

Intrauterine repair of myelomeningocele begins with intramuscular administration of fentanyl and vecuronium for fetal anesthesia. The repair is performed by an elliptical incision of the skin up to the fascia, around the neural plate, with release of the connection with the sac, and then it includes multilayer closure with the creation of myofascial membrane-lined flaps, which are sutured atraumatically in the midline above the neural plate, ensuring a watertight and anatomically protective cover. This technique prevents CSF leakage and exposure of the neural tissue to the amniotic fluid. In cases of insufficient skin closure, an elliptical-shaped graft is used. After the repair of the myelomeningocele, the uterus is closed in two layers and an epiploic flap is applied to the hysterotomy after first adjusting the amount of amniotic fluid to normal levels and suturing the fetal membranes to the myometrium. The laparotomy is closed in layers. Perioperatively, tocolytics (magnesium sulfate, indoquine, and nifedipine) are administered and fluids are restricted to prevent pulmonary edema. Patients are hospitalized postoperatively for approximately 4 days and are then monitored weekly, with delivery scheduled at 37 weeks [6,41].

#### 5.1.1. Complications of Open Intrauterine Surgical Repair of Myelomeningocele

The risk of preterm premature rupture of membranes, and as a result preterm labor, is related to smaller gestational age (<23 weeks) at the time of the intrauterine procedure. Furthermore, because the hysterotomy is performed in the uterine fundus, it is associated with an increased risk of uterine rupture or dissection [42].

#### 5.1.2. MOMS Study

To definitively evaluate the safety and efficacy of prenatal open myelomeningocele repair compared to the classic postnatal approach, the landmark MOMS (Management of Myelomeningocele Study) was implemented at three centers, Children’s Hospital of Philadelphia, University of California, San Francisco, Vanderbilt University Medical Center Nashville, and the study was coordinated by George Washington University, Washington, D.C. A total of 183 women with singleton pregnancies of 19.0–25.6 weeks and normal karyotype were randomized. The primary outcome of the study was the rate of intrauterine or neonatal death or the need for a ventriculoperitoneal drainage valve by 12 months of age, which was observed in 68% of the neonates in the prenatal surgery group versus 98% in the postnatal surgery group. Actual valve placement was significantly lower in the prenatal group (40% vs. 82%). In addition, rates of hindbrain prolapse and syringomyelia were reduced, while the absence of prolapse was observed in 36% of the prenatally operated patients compared to only 4% in the control group. However, more operations were observed due to cord clamping syndrome in the prenatal group. It also showed that mental development (based on the Bayley Mental Development Index) and the difference between functional and anatomical level of impairment at 30 months were significantly improved in the prenatal rehabilitation group. Additionally, in terms of long-term outcomes, it was found that infants in the prenatal surgery group had significantly better motor function relative to the anatomical lesion compared with those who underwent postnatal surgery, were twice as likely to walk without aids (42% vs. 21%), and had higher scores on motor scales (Bayley, Peabody), even with more severe lesions. Prenatal repair of myelomeningocele was associated with increased maternal and obstetric complications. Delivery occurred at a mean of 34.1 weeks vs. 37.3 weeks in the postnatal group (*p* < 0.001), with severe prematurity (<30 weeks) in 13%. Increased rates of PPROM, preterm labor, and oligohydramnios were observed. Specifically, PPROM is a major limitation, as it is reported in 80% of cases. This happens due to challenge in membrane closure post-surgery, mechanical disruption during trocar insertion, and fragility of the membranes due to inflammation. As a result, there is a need for improved surgical materials and sealing techniques. Maternal risks included pulmonary edema (6%) and the need for transfusion (9%). In 35% of cases, thinning or rupture was recorded at the site of the hysterectomy during childbirth [5].

### 5.2. Endoscopic Intrauterine Surgical Repair of Myelomeningocele

Following the findings of the MOMS study, research has focused on developing a safer, minimally invasive technique that may expand the group of eligible patients and provide therapeutic benefits with fewer complications and in a higher proportion of fetuses with myelomeningocele. Thus, after initial attempts to treat myelomeningocele with minimally invasive techniques failed due to technical difficulties and poor perinatal outcomes (e.g., fetal acidosis), the completely percutaneous and laparotomy-assisted techniques were developed [43].

In the purely percutaneous approach, laparotomy is not required; the trocars are inserted through the abdominal and uterine walls under ultrasound guidance using the Seldinger technique. Postoperative maternal pain is minimal, and tocolytic therapy is not usually required, even in the immediate postoperative period. The average gestational age at delivery is 33 weeks, but the PPROM rate is close to 80% [10].

In contrast, in the laparotomy-assisted approach, a wide surgical approach is required, the uterus is completely externalized, and the trocars are placed under direct vision through the uterine wall. Postoperative maternal pain is more severe and requires intensive analgesic management, but the average gestational age at delivery is 37 weeks [44,45].

Thomas Kohl developed the purely fetoscopic approach, introducing the use of partial carbon dioxide insufflation (PCI) to improve handling and visualization within the uterus, and was instrumental in alleviating the initial concerns about the risk of fetal acidosis and subsequent neurological damage from the use of CO_2_. More recently, Baschat’s group documented the absence of metabolic acidosis in fetuses undergoing PCI [8,46].

Subsequently, the Brazilian group applied the PCI technique with the addition of the innovative use of a biocellulose patch, achieving watertight closure without suturing the dura mater and reversing the posterior cerebral subsidence [47]. No complications related to acidosis were observed. The method was used by groups in Europe and the US with similar safety [43].

### 5.3. Fetoscopic vs. Open Intrauterine Surgical Repair of Myelomeningocele

Studies have shown that prenatal and postnatal outcomes in infants up to 12 months of age, as well as those older than 18 months, after prenatal embryoscopic surgical repair compared to open intrauterine surgical repair of myelomeningocele are similar [48]. The embryoscopic technique allows for the possibility of vaginal delivery and eliminates the risk of uterine rupture, thus protecting potential subsequent pregnancies from perinatal risks; however, the embryoscopic approach is accompanied by increased financial costs.

The optimal surgical technique for repairing the defect has not yet been fully defined, and its future optimization may lead to even better long-term results.

### 5.4. Conclusions

Open fetal surgery for myelomeningocele repair has been shown to significantly improve pediatric outcomes, but it is accompanied by procedural risks for both the mother and fetus.

Women with fetuses with myelomeningocele who meet established criteria for intrauterine repair should receive counseling regarding all available management options, including the possibility of prenatal in utero surgery.

Patients interested in in utero repair of myelomeningocele should be referred for further evaluation and counseling to a specialized fetal therapy center, which has the necessary expertise, interdisciplinary team, infrastructure, and specialized services, capable of providing detailed information regarding the surgical procedure and the intensive perinatal care it requires.

## 6. Congenital Diaphragmatic Hernia

Congenital diaphragmatic hernia (CDH) is a congenital anomaly in which there is partial or complete absence of the diaphragm, resulting in a continuous communication between the thorax and the abdomen. The incidence of congenital diaphragmatic hernia is approximately 1 in 4000 births, and in 85% the lesion is located on the left [49]. Although the pathogenesis of congenital diaphragmatic hernia has not been fully elucidated, it is believed to be the result of impaired retinoid signaling during intrauterine life, which usually manifests itself before the 10th week of gestation. During the course of fetal development, the diaphragmatic defect allows the displacement of abdominal viscera into the thoracic cavity, which leads to increased intrathoracic pressure. This pressure prevents the normal development of the airways and pulmonary vasculature and disrupts the normal respiratory movements of the fetus. The most important subsequent developmental and functional problems are pulmonary hypoplasia and pulmonary hypertension. Consequently, the condition is accompanied by a high risk of neonatal death due to respiratory failure and pulmonary hypertension. Surviving infants may present with serious complications, such as gastrointestinal and respiratory problems, orthopedic deformities, and neurodevelopmental delay [17].

### 6.1. Prenatal Diagnosis and Evaluation

Ultrasound is the main method of diagnosing congenital diaphragmatic hernia, with prenatal detection rates of up to 75% in specialized centers. In these fetuses, prenatal evaluation to assess potential postnatal survival is based on the presence of other significant anatomic and chromosomal abnormalities, measurement of lung size, and identification of intrathoracic liver herniation by ultrasound or MRI [50].

The lung-to-head ratio (LHR) is the most widely used prenatal marker of the severity of congenital diaphragmatic hernia, as it reflects the degree of pulmonary hypoplasia. Between 22 and 25 weeks of gestation, the typical four-ventricular projection of the heart is visualized on a two-dimensional ultrasound and is calculated as the lung area opposite the defect divided by the head circumference. An LHR <1.0 at 24 weeks of gestation is considered a high-risk predictor of perinatal mortality. Because LHR varies with gestational age, lung size is best represented as the ratio of observed-to-expected lung-to-head ratios (o/e LHR). Values <25% indicate that the fetus has severe pulmonary hypoplasia and are associated with high perinatal mortality and morbidity (probability of survival < 25%), while values >35% are associated with survival of up to 85% and a good prognosis [51,52].

### 6.2. Management of CDH

**Figure 2:**
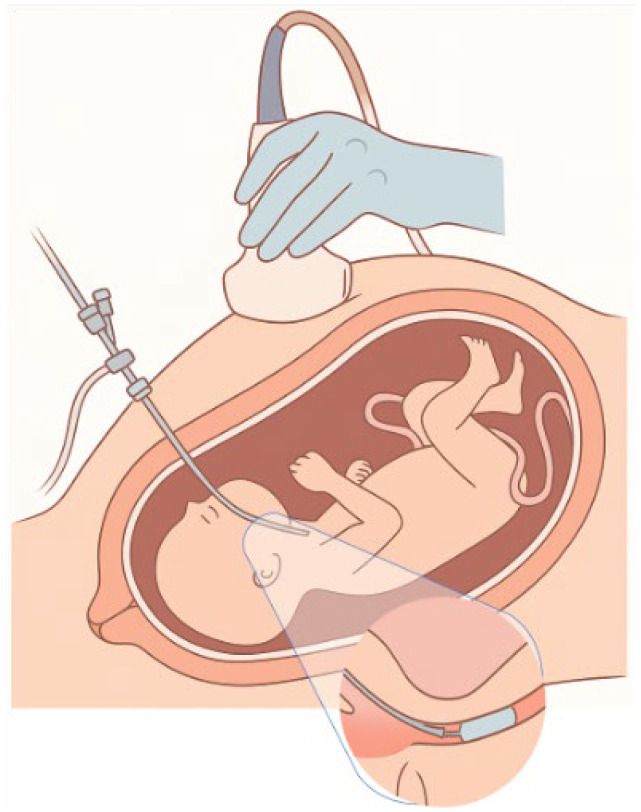
Fetoscopic tracheal occlusion.

The initial intrauterine interventions for the repair of congenital diaphragmatic hernia aimed at moving the liver into the abdominal cavity, a technique with serious complications, such as umbilical vein injury and intrauterine death. Subsequently, the recognition of the beneficial effect of tracheal occlusion on pulmonary development led to new, targeted therapeutic approaches. The first clinical application of tracheal occlusion was achieved by placing an external clip in 1993 by Harrison [18,53], while the mother was under general anesthesia. Later, the endoscopic technique of placing a detachable silicone or latex balloon in the trachea and filling it with saline was developed. This technique, known as Fetoscopic Endoluminal Tracheal Occlusion (FETO), shown in Figure 2, is performed between the 27th and 30th week of gestation in selected pregnancies with an unfavorable prognosis. The first step is the ultrasound-guided positioning of the fetus into the uterus in such a way that the oropharynx becomes distally accessible through the upper uterine half [54]. Achieving the desired fetal position is often difficult due to the limited possibilities of mobilization through external manipulations. Once the ideal position is achieved, the fetus is administered intramuscular anesthesia and muscle relaxation to immobilize it and minimize complications during the intervention. During the procedure, a 1mm embryoscope is inserted into the uterus and then into the fetus’s mouth, where a small balloon is placed and inflated in the trachea. During the procedure, a multidisciplinary team including fetal surgeons, anesthesiologists, pediatricians with airway expertise, and nurses is required in case of emergency delivery, where the balloon may cause obstruction of the newborn’s airway. In this case, the team must be ready to proceed with emergency balloon removal. The balloon is removed either by a second embryoscope or by ultrasound-guided removal approximately 4 to 6 weeks later (approximately at week 34). Insertion and removal can be performed with the mother under local anesthesia, and in some centers, combined spinal–epidural anesthesia is also applied to the mother [55,56].

To evaluate FETO, the Tracheal Occlusion to Accelerate Lung Growth (TOTAL) study was designed, which included 80 women (1:1 randomization), and showed that 40% of infants who underwent FETO survived to 6 months of age, compared with 15% in the conservative management group. Regarding complications, premature preterm rupture of membranes (PPROM) was recorded in 47% of women in the FETO group, compared with 11% in the control group, while preterm delivery occurred in 75% of cases in the FETO group (mean gestational age at delivery 34 weeks and 4 days) and in 29% in the conservative monitoring group (mean gestational age at delivery 38 weeks and 3 days). The mean birth weight in the FETO group was 481 g lower. The use of FETO showed an acceptable safety profile for the mother [9].

## 7. Lower Urinary Tract Obstruction (LUTO)

Congenital lower urinary tract obstruction is a rare congenital anomaly, with an estimated incidence of approximately 2.2 cases per 10,000 live births. The condition occurs more frequently in male fetuses than in female fetuses. The most common cause of LUTO is posterior urethral valves (PUV), which almost exclusively affect males. Less common causes include midurethral hypoplasia, anterior urethral valves, urethral stricture, ureterocele, urethral agenesis, and urethral strictures, which can also be seen in female fetuses with a clinical picture similar to that of PUV. In 5–8% of cases, LUTO is associated with chromosomal abnormalities [57].

LUTO is often accompanied by severe oligohydramnios, which can lead to lung hypoplasia and, ultimately, neonatal death. In addition, among the fetuses that survive, approximately 25–30% may have significant impairment of fetal renal parenchymal development, ultimately progressing to end-stage renal failure, requiring dialysis or kidney transplantation by the age of five years [11].

The diagnosis is usually made prenatally, during fetal anatomy ultrasound in the second trimester of pregnancy. Characteristic ultrasonographic findings include a megacystic appearance with a “keyhole” sign due to dilatation of the proximal urethra, hydronephrosis, and oligohydramnios, with or without cystic renal lesions. In the most severe cases, the diagnosis can be made as early as the first trimester of pregnancy. However, such prenatal findings are not sufficient to differentiate posterior urethral valves from other causes of congenital lower urinary tract obstruction, and definitive diagnosis is usually made after delivery. When the ultrasonographic appearance of this condition occurs between the 16th and 24th weeks of gestation, the prognosis is poor due to the increased incidence of pulmonary hypoplasia, which contributes significantly to the high perinatal morbidity and mortality [11].

Although correction of the obstruction after birth can restore urinary tract patency, it is usually not sufficient to prevent irreversible effects on the kidneys and lungs. Recognition of the serious renal and respiratory consequences of congenital lower urinary tract obstruction has led to the need to develop intrauterine therapeutic interventions [58].

### 7.1. Vesicoamniotic Shunt (VAS)

The most common and established method is the percutaneous vesicoamniotic shunt (VAS), which is performed under continuous ultrasound guidance and local anesthesia of the mother, with the placement of a shunt (such as Rodeck/Rocket or Harrison catheters) in the lower abdomen to facilitate bladder decompression. The distal end of the catheter is placed within the fetal bladder, while the proximal end ends in the amniotic cavity, allowing continuous drainage of fetal urine into the amniotic fluid. In cases of severe oligohydramnios, prior amnioinfusion may be required to increase the volume of amniotic fluid and create appropriate conditions for the safe placement of the shunt. This technique aims to decongest the fetal bladder and restore amniotic fluid volume, with the aim of improving lung development and preventing irreversible renal damage [59,60].

#### Complications of VAS

Despite its potential benefits, the method is not without complications, which are observed in up to 40% of cases. Displacement of the catheter is the most common complication, with a frequency reaching up to 40%. Ascites of genitourinary etiology is observed in approximately 20% of cases, while abdominal wall defects, such as gastroschisis, have been reported in up to 10% of fetuses. Premature birth is common in pregnancies with intrauterine intervention, which decisively affects the outcome. Survival rates range between 50% and 90%, depending on the severity of the initial pathology and the complications of the intervention. In approximately one third of cases, neonatal survival is determined by the functional adequacy of the renal parenchyma as well as the presence or absence of other serious congenital anomalies. However, the effect of the procedure on long-term renal function remains unclear, despite increased survival rates [61].

### 7.2. Serial Vesicocentesis

Serial vesicocentesis is a simpler but less effective interventional procedure that involves repeated percutaneous drainage of the fetal bladder with a thin needle under ultrasound guidance. This procedure provides temporary decompression and is used primarily in early pregnancy (<16 weeks) or when shunt placement is not feasible. Despite its simplicity, the technique requires multiple punctures and carries risks such as hemorrhage, infection, amniotic sac rupture, and premature labor. Its main role is diagnostic, as it provides the ability to collect urine for analysis (electrolytes, osmolality, β-2 microglobulin), in order to assess renal function and decide on the appropriate therapeutic strategy. Its therapeutic value remains limited, as it does not provide permanent restoration of urinary outflow [11].

### 7.3. Endoscopic Intrauterine Ablation of the Posterior Urethral Valves

In specialized centers, intrauterine endoscopic ablation of the posterior urethral valves using a fetoscope has also been experimentally applied. This technique, although extremely technically demanding, has the advantage of etiological treatment. However, its application remains limited, and further studies are required to evaluate its safety and efficacy [62].

## 8. Prenatal Management of Fetal Thoracic Malformations

### 8.1. Congenital Pulmonary Airway Malformation

Congenital Pulmonary Airway Malformation (CPAM) is a congenital lung anomaly that can manifest from fetal life to childhood. Although it is a rare entity, it is the most common form of congenital lung damage. Its incidence is estimated at approximately 1 per 4000 pregnancies [63]. CPAM is characterized histologically by the presence of multiple cysts within the lung, which result from hyperplasia and disruption of the formation of the terminal bronchioles, with the absence of normal alveoli. These cysts communicate with the normal bronchial tree, but they do not participate in the gas exchange process. In the majority of cases (over 95%), the damage is unilateral and affects a lobe or part of the lung [64]. The occurrence of fetal hydrops is recorded in less than 10% of cases, while hydramnios may occur later, usually after the 26th week of gestation, due to mechanical obstruction or compression of the esophagus by the thoracic mass. The pathogenesis of CPAM remains incompletely understood, although it is considered to be related to a disorder of the proximal–distal development of the respiratory epithelium during fetal life. This disorder is probably attributed to defective signaling between the respiratory epithelium and the underlying mesenchyme, leading to the absence of normal alveoli and the development of a polycystic mass. Regarding the accompanying abnormalities, no increased frequency of chromosomal or genetic syndromes has been found. However, in approximately 10% of cases, congenital anomalies of other systems are observed, with heart disease, renal malformations and tracheoesophageal fistulas being the most common [65].

Diagnosis is mainly made prenatally by ultrasound, usually after the 16th week of gestation, during which the lesion is visualized as an echogenic intrathoracic mass. Depending on its ultrasound appearance, CPAM is classified into macrocystic form (with cysts > 2 cm), microcystic (solid, without distinct cysts) and mixed form, which includes areas of both solid and cystic nature (cysts < 2 cm) [64].

Systematic evaluation of CPAM includes the choice between conservative monitoring, pharmacological intervention or surgical resection after birth or even intrauterine intervention in extremely severe cases. Intrauterine therapeutic intervention is considered when these lesions are accompanied by signs of compression, such as fetal hydrops, pleural effusion or hydramnios, as in these cases the development of the lung parenchyma is inhibited, increasing the risks of perinatal morbidity and mortality. Intrauterine intervention includes the placement of thoracoamniotic drainage [63]. The main therapeutic goal is to reduce intrathoracic pressure, in order to improve lung development and prevent or resolve hydrops. In rare cases with hydrops, open intrauterine resection of the lesion has been applied, with relatively satisfactory results. Intrauterine thoracocentesis of the fetus is applied for transient decompression of isolated cysts. However, in most cases, the contents of the cysts reaccumulate, making the method temporary and symptomatic and its use is limited. At the same time, there are indications, mainly in rapidly growing microcystic or solid lesions, that prenatal administration of corticosteroids can lead to lesion shrinkage and regression of hydrops [65].

Pregnancy monitoring is based on ultrasound examination every four weeks, with the aim of assessing the progression of the lesion, lung development and the amount of amniotic fluid. During the first half of the third trimester, more than 80% of microcystic lesions appear to regress ultrasound. However, in most of these cases, this is not a true regression of the lesion, but rather an ultrasound non-detectability, due to increased echogenicity and normal lung parenchyma. In these cases, the lesion can be detected postnatally by chest radiography or, more accurately, by computed tomography [63].

It is recommended that delivery be performed in a maternity hospital with neonatal intensive care and pediatric surgical support. The ideal time of delivery is at 38 weeks, unless there are signs of worsening fetal condition, such as growth retardation, hypoxia, or hydrops, in which case early induction of labor is indicated. The preferred method of delivery is vaginal delivery, provided there are no obstetric contraindications [66].

The prognosis is excellent in cases without hydrops, with survival rates exceeding 95%. Conversely, the presence of hydrops is an extremely unfavorable prognostic indicator and is associated with high perinatal mortality. Finally, no increased risk of recurrence of CPAM has been reported in [67].

### 8.2. Bronchopulmonary Sequestration

Bronchopulmonary sequestration is a rare congenital anomaly, with an estimated incidence of approximately 1 in 15,000 births. Prenatal diagnosis is mainly achieved through ultrasound, where an echogenic mass is observed within the pulmonary parenchyma, usually in the left lower lobe. The use of color Doppler is decisive, as it highlights a feeding blood vessel originating from the descending aorta [68].

In approximately 75% of cases, the effusion is intralobular, which creates difficulty in differentiating it from the microcystic form of CPAM [69]. On the contrary, in the remaining 25% it is extralobular, which is located outside the normal lung tissue and is surrounded by its own visceral pleura. In most of these cases, an accompanying pleural effusion is observed.

There is no documented increased risk of chromosomal abnormalities or genetic syndromes. However, in cases of extralobular effusion, in up to 50% of cases, other congenital anomalies coexist, mainly diaphragmatic hernia, as well as cardiac or spinal malformations [70].

Intrauterine therapeutic intervention includes ultrasound-guided endoscopic interruption of blood perfusion of the lesion using a laser, especially in cases of significant hydrothorax or fetal hydrops. Monitoring is performed every four weeks with ultrasound examinations, in order to assess the evolution of the size of the lesion and the accompanying findings. In more than 30%, a reduction or complete regression of the mass is observed during the third trimester of pregnancy [71,72].

It is recommended that delivery take place in a hospital with neonatal intensive care and pediatric surgical coverage. The recommended time point is 38 weeks of gestation, with planned induction of labor and the goal of vaginal delivery. The prognosis is excellent, with survival rates exceeding 95%. Postnatal management includes endoscopic removal of the lesion or selective embolization of the supplying vessel. There is no increased risk of recurrence [73].

### 8.3. Congenital High Airway Obstruction Syndrome

Congenital High Airway Obstruction Syndrome (CHAOS) is a rare, with a prevalence of 1 in 50,000 births, but often fatal fetal condition. It results from intrinsic obstruction of the fetal upper airway, most commonly due to laryngeal or tracheal atresia. The airway blockage prevents normal egression of lung fluid, leading to progressive bilateral lung enlargement, flattening or inversion of the diaphragm, hydrops fetalis, and ultimately cardiac failure, if left untreated. Prenatal diagnosis is typically made by ultrasound and confirmed by fetal MRI, which demonstrates enlarged echogenic lungs and a distended tracheobronchial tree. The standard approach to management in non-hydropic fetuses diagnosed in the second or third trimester is the ex utero intrapartum treatment (EXIT) procedure.

The EXIT procedure is a specialized intervention, performed during labor, that allows the preservation of placental blood flow to the fetus during its partial exit from the uterus. Its purpose is to surgically secure an airway of the fetus via intubation, bronchoscopy, or tracheostomy prior to complete delivery, before the umbilical cord is clamped, thereby preventing hypoxic injury at birth [74]. The successful implementation of the EXIT technique requires detailed preoperative planning and collaboration of a multidisciplinary team, which includes obstetricians, fetal and maternal surgeons, pediatric surgeons, maternal and fetal anesthesiologists, neonatologists, and specialized nursing staff. The preparation is based on imaging control with ultrasound, fetal heart ultrasound, magnetic resonance imaging, as well as genetic assessment and counseling, where required [74]. The procedure involves reducing myometrial activity through tocolysis and anesthesia, positioning the mother in an appropriate position, performing a hysterotomy in a way that ensures the integrity of the uterus, and the gradual exit of the head and upper torso of the fetus. The volume and temperature of the amniotic fluid are maintained throughout the intervention, in order to ensure visual adequacy and stable blood perfusion through the placenta. Intrauterine drugs for fetal anesthesia are administered and the fetal heart rate and oxygen saturation are continuously monitored. After the completion of the required procedures, the umbilical cord is ligated and treatment is administered to prevent uterine atony [74]. In hydropic cases diagnosed earlier in gestation, fetoscopic decompression of the airway has been explored as an investigational intervention, although it is associated with significant technical challenges and remains experimental [75]. Accurate diagnosis, multidisciplinary planning, and delivery at a specialized fetal surgery center are essential to optimize outcomes in affected pregnancies.

Despite its advantages, the EXIT technique is not without risks. Potential maternal complications include bleeding due to uterine atony or placental abruption, the need for hysterectomy, and an increased risk of uterine rupture in future pregnancies. Fetal and neonatal complications include death and severe neurodevelopmental disability. Compared with classic cesarean section, EXIT is associated with greater blood loss, prolonged operative time, and an increased risk of surgical complications [74,75].

Therefore, patient selection should be rigorous, based on evidence-based criteria, and preoperative counseling of parents should be detailed, including all potential complications and uncertainty about the final prognosis. The success of the technique depends largely on the experience of the team and the adequacy of the logistical infrastructure of the application center.

## 9. Future Applications of Prenatal Therapeutic Interventions

### 9.1. In Utero Gene Therapy (IUGT)

Prenatal fetal gene therapy is emerging as a promising approach for the treatment of single-gene diseases before birth. Its advantage lies in its early intervention, which can prevent the onset or progression of serious pathologies, such as neurodegeneration and heart failure, that appear already in fetal or neonatal life. In addition, the immature immune system of the fetus facilitates immunological tolerance to foreign antigens, reducing the risk of gene therapy rejection and allowing long-term expression of the therapeutic gene. Prenatal gene therapy may allow for the penetration of the blood–brain barrier and targeting of the central nervous system before irreversible damage is established. At the same time, the smaller size of the embryo allows a higher amount of viral vector to reach the cellular target, increasing the efficiency of gene transfer. Experimental data from models in mice, sheep, and non-human primates demonstrate the possibility of achieving immunological tolerance, long-term gene expression and phenotypic improvement in diseases such as thalassemia, spinal muscular atrophy, Angelman syndrome, Duchenne muscular dystrophy, and congenital blindness [76,77,78,79].

However, the application of IUGT is accompanied by significant challenges and potential risks, such as integration of genetic material into non-targeted cells, transmission of gene modification to subsequent generations and possible side effects in the mother through transplacental leakage. Despite ethical and regulatory concerns, the successful course of gene therapy in children and adults and the accumulation of strong preclinical data create the appropriate foundation for the future implementation of IUGT clinical trials. Such an endeavor requires multidisciplinary collaboration, rigorous preclinical documentation, thorough parental education, and careful ethical evaluation [80].

To date, the application of prenatal gene therapy in humans has been limited to interventions that do not involve direct gene modification but focus on embryonic cell transplantation and protein amplification. Specifically, the only form of prenatal gene intervention in humans includes three main applications in research and clinical settings: 1. Intrauterine enzyme therapy for lysosomal diseases, such as Pompe disease and other lysosomal storage disorders at UCSF [81,82]. The treatment is administered intravenously through the fetal umbilical vein, with the aim of preventing the intrauterine onset of pathological enzyme accumulation. 2. Intravenous hematopoietic stem cell transplantation for α-thalassemia major, also performed by the same research group at UCSF. This strategy aims to correct severe fetal anemia through early transplantation of xenogeneic hematopoietic cells, favoring their long-term establishment through fetal immune tolerance. 3. Intraamniotic protein infusion for the treatment of X-linked hypohidrotic ectodermal dysplasia, was successfully applied in two human fetuses at the University of Erlangen in Germany [83]. The study was in 2018 and is the first documented case of prenatal protein therapy in humans. Injecting the missing protein ectodysplasin-A into the amniotic fluid resulted in the restoration of sweat gland development after birth, with long-term clinical improvement. It is worth noting that none of these methods constitute classical gene modification through DNA integration or repair but are transient applications of cellular or protein intervention aimed at maintaining function or stabilizing the disease before birth. The above efforts lay the foundation for the safe transition to true intrauterine gene therapy in the future.

### 9.2. Minimally Invasive and Robotic-Assisted Fetal Surgery

Fetal surgery has made significant progress in the last two decades, with the transition from open intrauterine surgery to minimally invasive techniques, aimed at minimizing maternal and fetal morbidity. The most common methods include fetoscopic surgery, intrauterine drainage, and endoscopic laser photocoagulation, as mentioned above [84].

Advances in instrument technology have allowed the construction of smaller, more flexible endoscopes, with improved visual acuity and better navigation of the intrauterine space. At the same time, robotic-assisted fetal surgery is in an experimental stage, but it is expected to play a decisive role in the future. Robotic systems, such as the da Vinci Surgical System, have already been used in experimental models to perform precise surgical procedures on the placenta and fetus, with reduced tissue trauma and increased stability [85].

In addition, newer technologies, such as magnetically steerable catheters, are being investigated as a means to further increase precision and safety, allowing dynamic guidance in real time with reduced mechanical stress on maternal and fetal tissues [86].

The minimization of surgical risks and the possibility of early therapeutic intervention have defined a new era in prenatal care. Advances in robotics, 3D imaging, and surgical precision will soon allow for the personalized treatment of even more complex fetal anomalies, with significantly improved outcomes for neonatal and long-term health [87].

## 10. Maternal Outcomes

One of the main concerns in fetal surgery is maternal morbidity and varies substantially depending on the surgical approach. The overall rate of maternal complications during pregnancy was 20.9% for open fetal surgery and 6.2% for fetoscopic procedures. Severe complications, classified as Clavien–Dindo grade III to V, occurred in 4.5% and 1.7% of cases, respectively [88].

Open fetal surgery and minimally invasive fetal surgery presents intraoperative and postoperative complications. The intraoperative ones for open fetal procedures included placental abruption (1.28%), significant hemorrhage requiring immediate delivery (0.92%), and the need for blood transfusion (1.00%). Fetoscopic procedures showed lower rates of complications, 0.28% of placental abruption, and 0.27% of transfusions. Serious maternal events are rare and include maternal cardiorespiratory arrest and disseminated intravascular coagulation (DIC) [89].

Postoperative complications included chorioamnionitis or endometritis (4.13% in open and 1.45% in fetoscopic surgeries), pulmonary edema (4.32% vs. 0.63%), and sepsis. Notably, hysterectomy was required in isolated cases, including one following DIC after laser for TTTS and another after open MMC repair with prior cesareans. At delivery, the most common complications of open fetal surgery were uterine rupture, reported in 0.9%, and uterine dehiscence, reported in 3.7% of patients. Fetoscopic procedures do not present with such complications at delivery, which highlights their lower impact on uterine integrity compared to open procedures [89].

Long-term maternal outcomes revealed uterine rupture in 6.89% and dehiscence in 11.09% of pregnancies following open fetal surgery [89]. However, fetoscopic procedures do not present these complications during follow-up. These findings suggest a greater uterine morbidity associated with open surgical access, which shows the importance of close surveillance in subsequent pregnancies.

## 11. Limitations: Cost and Ethical Considerations

### 11.1. Cost

Fetal surgery, particularly open procedures, requires substantial costs, including preoperative imaging and counseling, the surgical procedure itself, hospitalization, and prolonged maternal-fetal monitoring. Nevertheless, for certain conditions, fetal intervention may reduce the severity of postnatal disease and, as a result, the associated lifelong medical expenses. For example, in utero repair of myelomeningocele decreases the need for ventriculoperitoneal shunting postnatally and improves motor outcomes, as it stops the progression of the disease, which could translate to reduced long-term healthcare and rehabilitation costs. Although fetal interventions are associated with increased short-term hospitalization, recent studies have shown that this may offer substantial long-term economic and quality-of-life benefits. Prenatal repair of myelomeningocele has been shown to reduce the incidence of several significant postnatal morbidities, such as hydrocephalus requiring cerebrospinal fluid diversion, Chiari II malformation, and lower-limb motor deficits. In utero management prevents these complications and as a result, it may decrease future neurosurgical procedures, reduce long-term dependency, and enhance the patients’ functional status [15]. Thus, it results in potential reductions in lifelong healthcare costs and special education needs. Further research is necessary in order to evaluate the cost-effectiveness of prenatal procedures by including the outcomes from the evolving minimally invasive techniques. Several economic models have demonstrated that prenatal surgery can be cost-effective, and under certain assumptions, even cost-saving, compared to postnatal repair, especially when considering quality-adjusted life years. Moreover, apart from the direct medical costs, fetal procedures also affect indirect societal costs, such as the costs needed for caregivers. The benefits of in utero repair should be weighed against increased maternal morbidity, higher rates of obstetric complications, longer hospital stays during pregnancy, and impact for future maternal health, such as uterine rupture and the need for elective cesarean deliveries. Furthermore, fetal surgery requires experience and training of maternal–fetal medicine specialists and fetal surgeons, forming of a multidisciplinary team, as well as having the necessary infrastructure, such as neonatal intensive care, in order to significantly affect both perinatal outcomes and healthcare expenses. Fetal surgery requires increased cost, specialized infrastractrue and multidisciplinary expertise, which limits its implementation in resource-limited settings. Cost-reduction strategies, including regionalization of care, international collaboration, and development of lower-cost techniques, may enhance accessibility without compromising outcomes.

### 11.2. Ethical Considerations

An important ethical matter about fetal surgery is that we have to deal with two patients, the mother and the fetus. Unlike most medical interventions, the procedure directly benefits the fetus but presents significant risks to the mother. This asymmetry raises concerns about informed consent, autonomy, and the appropriate threshold for maternal risk in procedures conducted for fetal benefit [90]. As a result, informed consent in fetal surgery must be detailed, and it should ensure that mothers fully understand the potential complications such as uterine rupture, preterm labor, and consequences for future pregnancies [91]. Also, the medical team should discuss with the patients the risks and benefits of the procedure and explain that they should not have unrealistic expectations regarding surgical outcomes.

Furthermore, there is no equity of access, as discussed above. Fetal surgery can be offered only at specialized centers with advanced resources and a trained medical team, limiting its availability to certain geographic or socioeconomic populations, which raises questions about justice and fairness in medical care [91].

In order to safely and ethically implement fetal procedures, a clear progression from research to clinical application is essential. Fetal therapy is intended to treat the fetus and its development relies on carefully designed studies and clinical trials to establish its safety and efficacy. Additionally, many fetal procedures, such as in utero gene therapy, are still in early stages, and their long-term outcomes for both the child and the mother remain uncertain. In utero gene therapy permanently modifies the fetal genome, and it raises ethical concerns about off-target effects and long-term consequences. Long-term follow-up of the mother and the child is needed in order to ensure their safety and efficacy.

## Figures and Tables

**Table 1:** Summary of the most common procedures—indications, techniques, and outcomes.

Conditions	Indications	Time	Techniques	Outcomes
Fetal anemia	MCA PSV exceeds 1.5 MoM or present hydrops caused by fetal anemia	18–35 weeks	Ultrasound-guided intrauterine transfusion	High survival rates if performed early; risk of preterm labor or fetal bradycardia
Twin-to-twin transfusion syndrome (TTTS)	stages II–IV	16–26 weeks	Fetoscopic laser photocoagulation of placental anastomoses	Survival of both twins: ~65% Survival of at least one twin: ~88%
Myelomeningocele	Open neural tube defect with hindbrain herniation and motor impairment risk	19.0–25.9 weeks	Open fetal surgery or fetoscopic repair	Improved motor function, reduced need for shunting; increased maternal risk
Congenital diaphragmatic hernia (CDH)	Severe pulmonary hypoplasia due to abdominal organs herniation into thorax	severe CDH: 27–29 weeks moderate CDH: 30–32 weeks Balloon removal is ideally performed at around 34 weeks	Fetoscopic Endoluminal Tracheal Occlusion (FETO)	In severe CDH, FETO significantly improved survival (40% vs. 15% in expectant group). In moderate CDH, FETO did not show statistically significant survival benefit but was associated with a higher rate of preterm birth and premature rupture of membranes.
Lower Urinary Tract Obstruction (LUTO)	- Confirmed diagnosis of bladder outlet obstruction, most commonly posterior urethral valves. - Evidence of progressive bladder distension and oligohydramnios, which threaten lung development. - Normal or mildly impaired renal function, typically assessed via fetal urine biochemistry (e.g., sodium < 100 mEq/L, chloride < 90 mEq/L, osmolality < 210 mOsm/kg). - No associated lethal anomalies or chromosomal abnormalities. - High risk of pulmonary hypoplasia if untreated, due to persistent low amniotic fluid volume.	18 and 24 weeks	Vesicoamniotic shunting (VAS)	VAS survival from 40% to 70% by preventing pulmonary hypoplasia, but renal outcomes remain a major limitation, and many survivors still require postnatal interventions or dialysis.
Congenital Pulmonary Airway Malformation (CPAM)	Large cystic lung lesion causing mediastinal shift or hydrops	18–28 weeks	Thoracoamniotic shunt	Good prognosis if hydrops resolves; risk of recurrence or preterm labor
